# Sleep Stage Classification Through HRV, Complexity Measures, and Heart Rate Asymmetry Using Generalized Estimating Equations Models

**DOI:** 10.3390/e26121100

**Published:** 2024-12-16

**Authors:** Bartosz Biczuk, Sebastian Żurek, Szymon Jurga, Elżbieta Turska, Przemysław Guzik, Jarosław Piskorski

**Affiliations:** 1 Institute of Physics, University of Zielona Góra, 65-069 Zielona Góra, Poland; bbiczuk@gmail.com (B.B.); s.zurek@if.uz.zgora.pl (S.Ż.); 2 The Doctoral School of Exact and Technical Sciences, University of Zielona Góra, 65-417 Zielona Góra, Poland; 3 Faculty of Medicine and Health Sciences, University of Zielona Góra, 65-069 Zielona Góra, Poland; s.jurga@cm.uz.zgora.pl; 4 Institute of Pedagogy, University of Zielona Góra, 65-417 Zielona Góra, Poland; e.turska@wpps.uz.zgora.pl; 5 Department of Cardiology—Intensive Therapy, Poznan University of Medical Sciences, 60-355 Poznań, Poland; pguzik@ptkardio.pl

**Keywords:** heart rate asymmetry, sleep stages, sample entropy, HRV, GEE, predictive machine learning

## Abstract

This study investigates whether heart rate asymmetry (HRA) parameters offer insights into sleep stages beyond those provided by conventional heart rate variability (HRV) and complexity measures. Utilizing 31 polysomnographic recordings, we focused exclusively on electrocardiogram (ECG) data, specifically the RR interval time series, to explore heart rate dynamics associated with different sleep stages. Employing both statistical techniques and machine learning models, with the Generalized Estimating Equation model as the foundational approach, we assessed the effectiveness of HRA in identifying and differentiating sleep stages and transitions. The models including asymmetric variables for detecting deep sleep stages, N2 and N3, achieved AUCs of 0.85 and 0.89, respectively, those for transitions N2–R, R–N2, i.e., falling in and out of REM sleep, achieved AUCs of 0.85 and 0.80, and those for W–N1, i.e., falling asleep, an AUC of 0.83. All these models were highly statistically significant. The findings demonstrate that HRA parameters provide significant, independent information about sleep stages that is not captured by HRV and complexity measures alone. This additional insight into sleep physiology potentially leads to a better understanding of hearth rhythm during sleep and devising more precise diagnostic tools, including cheap portable devices, for identifying sleep-related disorders.

## 1. Introduction

Sleep is a fundamental physiological process that occupies one-third of our lives. It is essential for maintaining overall health and well-being and is critical for maintaining health. Proper sleep is vital for cognitive functions, physical health, and emotional stability. Disrupted sleep can lead to a range of health issues including impaired cognitive function, mood disorders, and metabolic problems [1].

Accurate detection and differentiation of sleep stages are crucial for the diagnosis and management of various sleep-related disorders. Conditions such as insomnia, sleep apnea, circadian rhythm disorders, and hypersomnia can be identified through detailed sleep studies. For instance, insomnia is a prevalent issue in industrialized countries, affecting a significant portion of the population. Early detection and management of such sleep disorders can prevent their progression and mitigate associated health risks. Sleep studies help establish the diagnosis of these conditions by helping identify abnormalities in sleep patterns [2].

Sleep studies play a crucial role in the prevention of accidents caused by sleep deprivation. Lack of adequate sleep can severely impair cognitive and motor functions, leading to reduced productivity and increased risk of accidents. By understanding sleep stages and improving sleep quality, the overall productivity can be enhanced and the likelihood of accidents can be reduced [2].

Polysomnography (PSG) is the gold standard for sleep stage classification, providing comprehensive data on multiple physiological signals, such as brain activity (EEG), eye movements (EOG), muscle activity (EMG), and heart rate (ECG). Among these, the ECG signal and its derivative, the RR intervals time series, have garnered significant attention for their potential in sleep stage detection, because it is the easiest and cheapest signal to record, which at the same time has a multitude of methods for its analysis. There are many devices that can cheaply record the ECG and its derivative RR intervals time series.

### 1.1. Sleep Stages and Transitions

Sleep is a complex and dynamic process characterized by the cyclical occurrence of various stages. The stages of sleep are typically classified into non-rapid eye movement (NREM) stages (N1, N2, N3) and rapid eye movement (REM) stage (R), along with the wakefulness stage (W).

The N1 stage is the lightest stage of sleep, often considered the transition between wakefulness and sleep. This stage usually lasts for a few minutes, occurs multiple times during sleep, and accounts for about 5–10% of total sleep time [3]. The N2 stage is a deeper sleep stage and it accounts for approximately 45–55% of total sleep time. During this stage, the body continues to relax, and the heart rate and body temperature decrease [4,5]. The N3 stage, also known as slow-wave sleep (SWS) or deep sleep, is crucial for restorative processes, including tissue repair and growth. N3 sleep comprises about 15–25% of total sleep time and is most prominent in the first third of the night [5,6]. The R stage, or REM sleep, is characterized by rapid eye movements, low muscle tone, and vivid dreaming. The brain activity during REM sleep is similar to wakefulness. REM sleep accounts for about 20–25% of total sleep time and is essential for cognitive functions such as memory consolidation and learning [5]. The W stage represents wakefulness. It includes periods of being awake during the night, often interspersed between sleep cycles [5].

Transitions between these stages are essential for understanding sleep architecture. For example, the transition from N3 to N2 indicates a lightening of sleep, often occurring before entering REM sleep or waking briefly [4]. Direct transitions from N3 to REM sleep are rare, typically requiring an intermediate stage; when they occur, they indicate a significant shift in brain activity from restorative processes to cognitive functions [6]. Transitioning from REM sleep to N2 is common and indicates the end of a REM period, usually cycling back to N2 before progressing through other sleep stages [5].

Sleep stages and transitions are usually classified by a trained person on the basis of the EEG recording—other signals available in the polysomnographic recording are also helpful for this task [6].

### 1.2. Sleep Stage Alterations During Various Disease Conditions

The subjects in this study suffer from various diseases, necessitating consideration of sleep stage alterations associated with their conditions.

Sleep stage alterations are frequently observed in disease states, disrupting sleep architecture and quality. For instance, obstructive sleep apnea (OSA) results in intermittent hypoxia and frequent arousals, significantly reducing restorative slow-wave sleep (N3 stage) and REM sleep. In OSA, apnea episodes elevate systemic and pulmonary pressures during sleep, with blood pressure surges particularly noted during REM stages, contributing to cardiovascular strain and hypertension progression [7,8,9]. Hypertension commonly co-occurs with OSA, and apneic events activate the sympathetic nervous system, contributing to sustained high blood pressure during wakefulness as well [10,11].

Cerebrovascular conditions like ischemic stroke and transient ischemic attacks (TIA) often show increased apnea-hypopnea index (AHI) and fragmented sleep, suggesting sleep-disordered breathing may worsen stroke outcomes through impaired nocturnal blood pressure regulation and reduced deep sleep stages [12,13,14]. Additionally, metabolic conditions such as diabetes and obesity are linked to reduced sleep efficiency and decreased N3 stage, with diabetic patients experiencing more arousals and sleep interruptions due to hyperglycemia-related discomfort and altered autonomic regulation [15,16].

### 1.3. Detecting and Classifying Sleep Stages on the Basis of ECG

The RR intervals reflect the heart’s beat-to-beat variability, which is influenced by the autonomic nervous system and closely related to sleep architecture [14,17]. Monitoring these intervals can provide valuable information about transitions between different sleep stages, including REM and NREM sleep [14,18]. The ability to detect sleep stages based solely on RR interval time series would be of significant value, considering the ease of measurement and recording, along with the availability of numerous analytical methods for their examination.

#### 1.3.1. Heart Rate Variability

Heart Rate Variability (HRV) is a key marker for understanding autonomic nervous system (ANS) dynamics across sleep stages, reflecting the interplay between sympathetic and parasympathetic branches. HRV patterns vary with sleep stages, corresponding to different levels of autonomic activity. Typically, the low-frequency (LF) component is associated with both sympathetic and parasympathetic activity, while the high-frequency (HF) component predominantly to parasympathetic (vagal) activity [17]. This approach allows interpretation of HRV results in terms of ANS branches; for instance, in NREM sleep, HRV spectra show higher HF and lower LF components, whereas REM sleep is characterized by increased LF/HF ratios [14].

Specific HRV patterns indicating high parasympathetic and low sympathetic activity are generally linked to better sleep quality, especially deep sleep (N3 stage) [18]. Ichimaru et al. investigated how sleep stages affect the relationship between respiration and HRV, finding that parasympathetic activity decreases during REM sleep compared to NREM sleep [19].

Herzig et al. assessed the reproducibility of HRV parameters across sleep stages, discovering that heart rate and LF power are highly reproducible in slow-wave sleep, while HF power and the LF/HF ratio vary significantly [20]. Tsunoda et al. examined light intensity effects on HRV, revealing that the LF/HF ratio increases under bright light or extreme darkness compared to dim light. They also found that LF power is higher during REM sleep, whereas HF power increases across all sleep stages, leading to a decreased LF/HF ratio in SWS [21].

The relationship between sleep stages and SDNN has been explored in various studies. Studies have suggested that SDNN can indicate overall autonomic activity during sleep. For instance, SDNN vary between sleep stages, with specific decreases in deep sleep (N3) and increases in REM sleep, which reflect shifts in autonomic balance [14]. The increase of SDNN during REM sleep may be attributed to a more variable autonomic activity [2].

Sleep stage classification using HRV metrics, including SDNN, has been explored in machine learning models. Classification models using HRV features from ECG data have shown that SDNN is a key parameter for distinguishing between wakefulness, REM, and NREM sleep [22]. Another study evaluated the accuracy of sleep stage classification using HRV features, revealing that SDNN and other HRV-based parameters offer a reasonably high level of accuracy in differentiating stages [23].

HRV measured across different sleep stages reflects disease and physiological states. For example, age significantly impacts HRV during sleep. Older individuals tend to exhibit lower HF components during NREM sleep compared to younger individuals, indicating reduced parasympathetic activity with aging [14]. Individuals with OSA show distinct HRV patterns, such as increased HRV during apneic events due to heightened sympathetic activity [1].

#### 1.3.2. Complexity

Studying complexities in RR interval series is an approach different from standard HRV analysis. A frequently used complexity parameter is Sample Entropy (SampEn) [24]. Several studies have examined the relationship between sleep stages and SampEn of RR intervals. Particularly in patients with sleep apnea, Sample Entropy is effective in capturing the physiological transitions between different sleep stages. Sleep-to-wake transitions exhibited a significantly larger decrease in SampEn compared to transitions within sleep stages. This reduction in SampEn was more pronounced than changes detected by HRV measures [25,26].

Both HRV and SampEn are correlated with various physiological and pathological findings, reflecting sympathetic activity or respiratory physiological control mechanisms. Subjects with OSA demonstrated a reduced parasympathetic activity and decreased SampEn compared to those without OSA, indicating an imbalance between sympathetic and parasympathetic modulation [27].

Patients with obstructive sleep apnea syndrome (OSAS) showed significant associations between complexities of the RR interval series and autonomic nervous activities, providing insights into the severity and impact of the disease [28]. SampEn of OSAS patients tend to have a more regular (less complex) pattern compared to normal individuals. This regularity was linked to the cyclic changes in heart rate caused by episodes of apnea and subsequent arousal, reflecting disruptions in autonomic function. In functional neuroscience, SampEn serves as a criterion for sleep staging, with lower entropy values observed during deeper sleep stages [29]. The addition of entropy-based regularity parameters has also been shown to improve sleep stage classification based on HRV using artificial neural networks for distinguishing between wakefulness, NREM, and REM sleep [30].

#### 1.3.3. Heart Rate Asymmetry

Heart rate asymmetry (HRA) is a novel approach to studying sleep and sleep stages, encompassing both variance-based and runs-based (microstructure) aspects. Variance-based HRA quantifies the different contributions of heart rate decelerations and accelerations to RR interval variability, highlighting that decelerations contribute more to short-term variability, while accelerations have a larger impact on long-term and total variability [31]. Runs-based HRA examines sequences of monotonic runs of accelerations and decelerations, revealing that accelerations tend to form longer and more numerous runs than decelerations [32].

Guzik et al. investigated the relationship between sleep stages and HRA, focusing on monotonic runs, in patients with OSA [33]. They found that decelerations and accelerations contribute unequally to heart rate variability (HRV), and this asymmetry is influenced by OSA severity. Heart rate data were analyzed to quantify HRA microstructure by measuring monotonic runs of decelerations and accelerations.

The results showed that patients with severe OSA had significantly fewer deceleration and acceleration runs of length 1 compared to those with moderate or no/mild OSA. Conversely, severe OSA patients exhibited a significantly increased number of longer runs (accelerations of length 5–10 and decelerations of length 5–8). Additionally, the longest acceleration runs were notably longer in the severe OSA group compared to subjects with no/mild OSA. The study concludes that HRA microstructure correlates with OSA severity, with severe OSA associated with a higher occurrence of longer deceleration and acceleration runs.

Jiang et al. explored the patterns of heart rate acceleration runs and deceleration runs in patients with OSA [34]. The study revealed that OSA significantly influences these patterns, indicating that the heart rate response to apnea events is characterized by asymmetric ARs and DRs [34].

#### 1.3.4. Complications of Using the RR Intervals Time Series Alone for Sleep Classification

The RR intervals time series and its derivative measures like HRV, complexity or HRA, are much more available and cheaper than full polysomnographic records, so these approaches generate a lot of interest since they could potentially be implemented in small, cheap, portable devices or even mobile phones. Recent advancements in wearable technology have made it possible to monitor HRV continuously throughout the night, offering new opportunities for long-term sleep studies and personalized sleep health management. Devices like the Oura ring have shown high accuracy in HRV measurement compared to medical-grade ECG, proving their utility in everyday sleep monitoring and lifestyle management [35].

Several studies have explored the use of HRV alone for sleep stage classification. This is a more difficult task, because less data are available. It can be expected that the complexity and HRA methods suffer from the same complications as HRV.

However, classifying sleep stages using only heart rate variability, complexity or asymmetry is challenging due to the intricate CNS–ANS interactions during sleep. As highlighted in [36], HRV does not fully capture the nuanced coupling between these systems, and sleep stages identified by EEG may not directly correlate with HRV patterns. Thus, while HRV provides insights into autonomic regulation, it may not reliably indicate EEG-defined sleep stages. This was further borne out by Stein et al. [14], who demonstrated that while general HRV patterns, such as decreased heart rate and variability during sleep stages 1–3 and increased values during REM sleep, can be identified, these relationships are complex and influenced by factors like age and clinical conditions. For example, older adults in the Sleep Heart Health Study did not show the typical increase in LF/HF ratio during REM sleep, and post-MI patients exhibited an increase rather than a decrease in LF/HF ratio moving from wake to non-REM sleep.

Also, it should be noted that while ECG recordings reflect changes in the autonomic nervous system during sleep, they do not provide a clear classification of sleep stages, even when complemented with respiratory signals. As noted in [37], these physiological signals are influenced by sleep stages, but precise identification relies on additional data like EEG recordings.

The aim of this paper is to investigate the relationship between HRA and sleep stages, a topic that has not been extensively explored due to the novelty of using HRA in sleep studies. To provide a comprehensive analysis, the study will also benchmark the results against other well-established parameters traditionally used to study the relationship between RR interval time series and sleep stages. These parameters include the SDNN, LF/HF, and SampEn. By comparing the asymmetric descriptors of heart rhythm with these conventional measures, the study aims to highlight the unique insights provided by HRA and its potential application in sleep research.

The rationale for taking up the task of detecting sleep stages on the basis of the RR intervals is the following. As we have already mentioned in the introduction, there are many devices that can record the ECG signal and derive and analyze the RR intervals much more cheaply than polysomnography. A method allowing for the detection of sleep stages without the use of EEG would provide a less invasive, more accessible, and potentially cost-effective approach for sleep monitoring, enabling broader applications in clinical and home settings.

## 2. Materials and Methods

Below, we present the data used in the present study and the methods for their analysis.

### 2.1. Group Description

The study included a group of 31 individuals (11 females) with various medical conditions, primarily focusing on sleep disorders. The ages of participants ranged from 27 to 84 years, with a median age of 58 years.

Participants presented with a range of comorbid conditions. The most common conditions were:

- Hypertension: 8 subjects;
- Obstructive Sleep Apnea (OSA): 5 subjects;
- Sleep Apnea: 12 subjects;
- Sleep Disorders: 11 subjects;
- Diabetes: 2 subjects;
- Ischemic Stroke: 1 subject;
- Transient Ischemic Attack (TIA): 2 subjects;
- Obesity: 1 subject;
- Asthma: 1 subject.

All volunteers gave informed consent to use the results of the polysomnographic recording in scientific research. According to the statement posted on the official website of the Collegium Medicum of the University of Zielona Gora this type of retrospective study that does not influence routine patient care does not require an explicit consent of the Committee [38].

### 2.2. Data Collection

The data for this study were obtained from polysomnographic recordings, specifically from the ECG channel. The specific device used was Comet PSG Plus XL (Grass Technologies, West Warwick, RI, USA). The data were exported to EDF files, then the ECG signal was extracted from each recording. The QRS complexes were first automatically annotated, then a trained technician reviewed each ECG recording to check the correctness of the automatic annotation and correct it if necessary. The in-house, open-source software, Signalweaver, was used for both tasks [39].

#### Data Segmentation and Sleep Stage Assignment

For each subject, the original recordings were annotated by a sleep expert who annotated each sleep stage in a continuous manner. For the purpose of this study, each recording was divided into 5-min segments to facilitate more granular analysis. In each segment, all the parameters analyzed in this paper were calculated, thus producing a dataframe with the number of segment as the row name and the parameter names as columns. Columns for each sleep stage were added to this dataframe. If a 5-min segment was entirely within a single sleep stage, this segment was assigned the value of 1, while if two or more sleep stages occurred within a segment, each sleep stage was assigned a fraction of 1, corresponding to the proportion of the segment occupied by the stage, rounded to one decimal point. Then, stage transitions columns were added and the sleep stages and transitions were cast to binary values of either 1 or 0 according to the following rules.

1. Certain Stage Detection: Certain stages (e.g., certain N2, certain N1) were identified for segments where the stage value was greater than a specified threshold (i.e., for fraction of 0.7 and more).
2. Detection of Transitions: For each recording, transitions between sleep stages were identified based on the following conditions:
  - A transition is marked if both the starting and ending stage columns are both non-zero for the analyzed segment, the ending stage value is less than or equal to a threshold (0.6), and the ending stage value does not decrease from the previous value (otherwise this would overwrite the opposite direction);
  - A transition is also marked if the starting stage column is non-zero and the corresponding element of the ending stage column is zero, but the next element in the ending stage column is non-zero and less than or equal to the threshold.

These methods were applied to the dataset to create new columns indicating certain stages and transitions, facilitating the classification of sleep stages.

### 2.3. HRV

Below, we will describe the HRV measures used to benchmark the performance of HRA measures, in both time and frequency domains.

#### 2.3.1. Time Domain

Among the parameters used to study HRV, the Standard Deviation of RR intervals (SDNN) is a key measure. SDNN represents the standard deviation of the RR intervals, taking into account only those of sinus origin (i.e., normal-to-normal heartbeats, hence NN in SDNN) and provides an overall assessment of HRV.

The descriptor SD1 is defined as the standard deviation of the distances of the points from the identity line in the Poincaré plot, which represents the short-term heart rate variability (HRV).

The descriptor *SD*2 is defined as the standard deviation of the points along the identity line in the Poincaré plot, which represents the long-term heart rate variability (HRV).

#### 2.3.2. Frequency Domain

The low-frequency component of HRV typically ranges from 0.04 to 0.15 Hz and is derived from the power spectral density of the RR intervals [40]. The high-frequency component ranges from 0.15 to 0.4 Hz [41].

To assess the balance between sympathetic and parasympathetic activity, the LF/HF ratio is used. This ratio is calculated as:

(1) $$LF/HF=\frac{LF}{HF}.$$

A higher LF/HF ratio is typically attributed to a dominance of sympathetic activity, whereas a lower ratio is claimed to suggest parasympathetic predominance [42].

### 2.4. HRA Variance Based Descriptors

Variance-based heart rate asymmetry (HRA) descriptors originate from the decomposition of HRV descriptors such as $SDNN^{2}$, $SD1^{2}$, and $SD2^{2}$ into components solely dependent on either decelerations or accelerations.

According to [43], $SD1^{2}$ can be divided into parts based on decelerations and accelerations as follows:

(2) $$SD1^{2}=SD1_{d}^{2}+SD1_{a}^{2}.$$

Similarly, it has been shown in [31] that long-term variability can be decomposed as:

(3) $$SD2^{2}=SD2_{d}^{2}+SD2_{a}^{2},$$

and the total variance can be expressed as:

(4) $$SDNN^{2}=SDNN_{d}^{2}+SDNN_{a}^{2}.$$

For detailed numerical methods and algorithms, refer to [31,43].

### 2.5. HRA Microstructure Descriptors

A run is an unbroken sequence of RR intervals that consistently shortens (heart rate accelerates), consistently lengthens (heart rate decelerates), or remains unchanged. This sequence is bordered by runs of different types. It is evident that this partitioning is unambiguous [32]. For a more detailed definition of runs, see [32].

The most straightforward descriptor is the count of runs of a particular type. Thus, $DR_{i}$ denotes the number of deceleration runs of length *i*, while $AR_{i}$ represents the number of acceleration runs of length *i*. Furthermore, $DR_{MAX}$ is the length of the longest deceleration run in a recording, and $AR_{MAX}$ is the length of the longest acceleration run in the recording.

As described in [32], the Shannon entropy [44], associated with the distribution of deceleration and acceleration runs, is partitioned into components that depend solely on decelerations or accelerations, namely $H_{D}$ and $H_{A}$. The mathematical details, physiologic properties, and applications can be found in [32,33,34,45,46,47].

It is important to also notice that there are other approaches to HRA, the most notable of which has been presented in [48,49]. This is a unique perspective on short-term heart rate asymmetry, emphasizing its connection to autonomic regulation. By employing time irreversibility analysis, the approach highlights temporal asymmetries in short-term heart period variability and links these patterns to autonomic nervous system dynamics. It can be said that in a way, this approach combines the variance based and structural parameters presented above.

#### Symmetric Count-Based and Asymmetric Threshold-Based Count Descriptors

These metrics are derived from the percentage of successive RR intervals that exceed specified thresholds. A number of symmetric count-based and asymmetric threshold-based count descriptors have been defined in [50]. In this paper, we will only use pNN30, $pNN30_{dec}$ and $pNN30_{acc}$, because both their similarities to the standard pNN50 parameter and their performance are shown in [50].

For further information and detailed mathematical derivations, refer to [50].

### 2.6. Sample Entropy (SampEn)

Sample Entropy (SampEn) is a statistical measure used to quantify the complexity or unpredictability of time-series data. It is widely utilized in physiological signal analysis, such as heart rate variability. SampEn improves upon Approximate Entropy (ApEn) by reducing bias and avoiding self-matches during calculation [24].

A detailed examination of these formulas may be found in [24]. In this paper, we used the very fast norm-component algorithm for SampEn calculation, described in detail in [51].

### 2.7. Generalized Estimating Equations (GEEs) for Logistic Regression

Generalized Estimating Equations (GEEs) are a statistical technique for analyzing clustered or correlated data, which is common in many biological and medical research studies. In this study, we applied GEE to perform logistic regression on 31 recordings, each consisting of multiple segments. Each recording (belonging to a single subject) serves as a cluster, and the segments within each recording are the elements of these clusters that need to be classified.

#### 2.7.1. GEE Approach

GEE extends the generalized linear model (GLM) to account for the within-cluster correlation of the response variables. This is achieved by specifying a working correlation matrix R(α), which models the correlation between the segments within the same recording. The parameter α encapsulates the strength and structure of this correlation. For mathematical details, see [52].

#### 2.7.2. Inference and Interpretation

The primary advantage of GEE is its robustness to the misspecification of the working correlation matrix. It provides consistent estimates of the regression coefficients β even if the assumed correlation structure is incorrect, provided that the mean model is correctly specified [52]. This robustness is particularly valuable in our study, where the exact nature of the within-recording correlation is complex and unknown.

#### 2.7.3. Prediction Using GEE

After constructing the GEE model, it can be utilized for prediction, thereby functioning as a machine learning method. The estimated regression coefficients $\hat{\beta }$ can be used to predict the probability of a new segment *j* within a new or existing recording *i* belonging to the target class.

The predictive performance of the GEE model can be evaluated using standard metrics such as accuracy, precision, recall, F1 score, and the area under the receiver operating characteristic curve (AUC). In our study, the GEE model was trained on a subset of the data and validated on the remaining data to assess its predictive capability.

#### 2.7.4. Application to Segment Classification

In our study, the GEE approach was employed in two distinct applications for segment classification: a purely statistical approach and a predictive approach for sleep stages and transitions between them.

#### Statistical Exploration of Sleep Stages

In the first application, we utilized GEE to explore the relationship between various independent variables and sleep stages, as well as transitions between these stages. The covariates $X_{ij}$ included features extracted from the RR intervals time series, pertaining to heart rate variability, heart rate complexity, and heart rate asymmetry.

The logistic regression model specified within the GEE framework enabled us to estimate the effects of various covariates on the occurrence and transitions between different sleep stages. By applying a backward selection process, using the Akaike Information Criterion (AIC) as the criterion, we systematically identified the most significant variables influencing these transitions. The GEE approach accounted for the within-subject correlation inherent in repeated measures, providing estimates of the regression coefficients.

#### Predictive Modeling of Sleep Stages

Following the statistical exploration, we employed the GEE models for predictive purposes. After building the models based on the statistical approach, we used them to predict sleep stages and transitions between them. The prediction involved estimating the probability of each segment belonging to a specific sleep stage using the fitted GEE model.

To evaluate the predictive performance of the GEE models, we used common machine learning metrics, including accuracy, precision, recall, F1 score, and the area under the receiver operating characteristic curve (AUC). These metrics allowed us to assess how well the models generalized to unseen data.

We believe it is possible to develop a predictive model using other techniques, such as multilayer neural networks, but they would not account for clustering as GEE does and would not enable us to perform statistical analysis.

### 2.8. Dataset Split and Model Type Selection

To ensure our predictive model accurately reflects the hierarchical structure of the data and maintains proportional representation of sleep stages, we divided the recordings into training and test sets using a stratified sampling approach. We began by calculating the distribution of sleep stages across the entire dataset and within each subject. Based on these distributions, we grouped the subjects into strata with similar sleep stage characteristics. From each stratum, we randomly assigned approximately 70% of the subjects to the training set and the remaining 30% to the test set—the result was that 71% of the data ended up in the training set, and 29% in the test set. This approach ensured that each sleep stage was proportionally represented within each cluster (subject) and across the resulting datasets. By assigning all data from each subject exclusively to either the training or test set, we preserved intra-subject correlations and maintained independence between the sets.

Out of the possible model types that are available for GEE [53], we selected the one-versus-all approach because it allows us to model each sleep stage separately and understand its unique associations with predictor variables.

### 2.9. Variables Entering the Analyses

The data are limited, so a selection of variables (features) is necessary. The following variables were selected for further analyses:

Symmetric variables: SDNN, SD1, SampEn, and LF/HF. These variables represent measures of variability and complexity in physiological signals and are considered important for understanding the dynamics of sleep stages. Another symmetric variable used in the analysis was pnn30, which is closely related to the widely used pnn50 [17,50].

Asymmetric Variables: $SD1_{a}$, $SD1_{d}$, $pnn30_{dec}$, $pnn30_{acc}$, AR4, DR4, $AR_{MAX}$, $DR_{MAX}$, $H_{DR}$, and $H_{AR}$.

All the selected metrics were calculated with the use of the free HraExplorer software v. 1.54 (https://hraexplorer.com/) in accessed on 30 August 2024. 5 min windows corresponding to the windows used to segment the sleep stages data. The source code for the software is available at [54] (frontend) and [55] (numerical library).

## 3. Results

This section presents the distribution of sleep stages and transitions, parameter variations across stages, and the outcomes of statistical models. It also includes an evaluation of predictive models for sleep stage classification and transitions, highlighting key metrics and findings.

### 3.1. Descriptive Statistics—Sleep Stage Distribution

In this subsection, we present the distribution of sleep stages and the transitions between them. We illustrate the frequency and percentage of each stage and transition observed across all recordings and within individual subjects, providing a foundational understanding of the data’s overall structure and variability.

The distribution of sleep stages and transitions between them is presented in the table below (Table 1).

**Figure 1:**
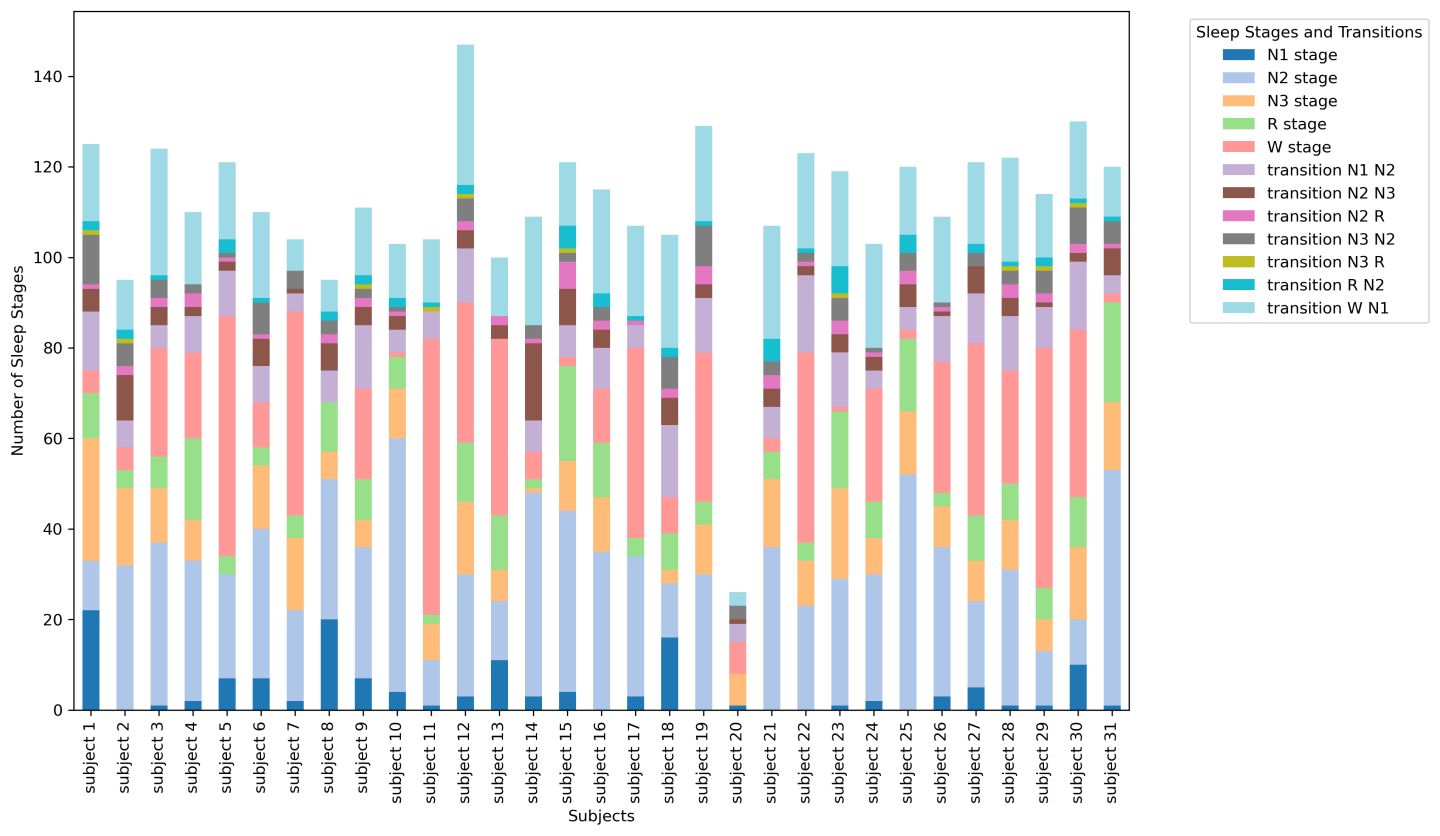
Distribution of sleep stages and transitions within individual subjects.

The distribution of sleep stages and the transitions between them within the recordings of individual subjects is presented in Figure 1.

### 3.2. Statistical Analysis

This subsection details the results of the Generalized Estimating Equation (GEE) regression analyses for various sleep stage transitions. We build the best models using backward selection with the AIC as the criterion. We identify significant predictors and assess their impact on the likelihood of transitions, providing insights into the statistical relationships between different sleep parameters and transitions.

We provide the results for individual values, but do not provide a *p*-value for the whole models, since this is not typically available for GEE—QIC or AIC is used for model comparison instead [52]. Deviance or pseudo-$R^{2}$ is not provided, as we do not see the utility for these parameters in the present study.

In what follows, whenever we talk about the *p*-value or a coefficient for a single variable, it should be understood that all other variables are kept constant. We believe that repeating this phrase every time we talk about a variable would distract from the message we are trying to convey.

#### 3.2.1. N1 Stage

Table 2 presents the results of a Generalized Estimating Equation (GEE) regression analysis for the binary response variable describing the N1 sleep stage. This is the best model selected with the use of backward selection on the basis of the AIC.

Symmetric Variables: In the N1 stage analysis, both SDNN and SampEn are statistically significant predictors with negative coefficients (−0.0236 for SDNN at p=0.007, and −0.4135 for SampEn at p=0.010). This indicates that higher values of these variables reduce the probability of a segment being classified as the N1 stage.

Asymmetric Variables: Among asymmetric variables, only $SD1_{a}$ is statistically significant (p=0.007) with a positive coefficient (0.0322), suggesting that an increase in $SD1_{a}$ raises the likelihood of identifying the N1 stage. Other asymmetric variables in the model were $pnn30_{\mathrm{acc}}$, $DR_{\mathrm{MAX}}$, and $H_{DR}$.

#### 3.2.2. N2 Stage

Table 3 shows results of a Generalized Estimating Equation (GEE) regression analysis for the binary response variable corresponding the N2 stage—best model.

Symmetric Variables: In the N2 stage analysis, LF/HF is a statistically significant predictor with a positive coefficient (0.0835, p<0.001), indicating that higher values significantly increase the probability of a segment being classified as the N2 stage. Other symmetric variables in the model were SDNN, SD1, pnn30, and SampEn.

Asymmetric Variables: Among asymmetric variables, DR4 and $AR_{\mathrm{MAX}}$ have statistically significant negative coefficients (−0.1330 for DR4 at p=0.003, and −0.2090 for $AR_{\mathrm{MAX}}$ at p=0.001), indicating that higher values reduce the probability of N2 stage identification. Additionally, $H_{DR}$ and $H_{AR}$ are statistically significant predictors with positive coefficients (1.2553 for $H_{DR}$ at p=0.039, and 0.4440 for $H_{AR}$ at p=0.006), suggesting that higher values increase the likelihood of identifying the N2 stage. The asymmetric variable $SD1_{d}$ was also included in the model.

#### 3.2.3. N1–N2 Transition

Table 4 shows the results of a Generalized Estimating Equation (GEE) regression analysis for the binary response variable describing the N1–N2 transition—best model.

Symmetric Variables: In the N1–N2 transition analysis, SDNN is a statistically significant predictor with a positive coefficient (0.0088, p=0.015), indicating that higher values increase the probability of transitioning from N1 to N2.

Asymmetric Variables: Among asymmetric variables, $H_{AR}$ is statistically significant with a positive coefficient (0.4436, p=0.024), suggesting that higher values significantly increase the likelihood of the N1–N2 transition. The asymmetric variable $SD1_{d}$ was also included in the model.

#### 3.2.4. W–N1 Transition

Table 5 presents the results of a Generalized Estimating Equation (GEE) regression analysis for the binary response variable describing the W–N1 transition—best model.

Symmetric Variables: In the W–N1 transition analysis, SDNN is a statistically significant predictor with a positive coefficient (0.0135, p<0.001), indicating that higher values significantly increase the probability of transitioning from wakefulness to N1 sleep. The symmetric variable SD1 was also included in the model.

Asymmetric Variables: No asymmetric variables were statistically significant in this analysis. Other asymmetric variables in the model were $SD1_{a}$, $SD1_{d}$, $AR_{\mathrm{MAX}}$, and $H_{AR}$.

#### 3.2.5. N2–N3 Transition

**Table 6:** GEE regression results, N2–N3 transition.

	Coef	Std Err	z	*p* > |z|	[0.025, 0.975]
Intercept	−3.5729	0.476	−7.502	0.000	[−4.506, −2.639]
SDNN	−0.0150	0.005	−3.103	0.002	[−0.025, −0.006]
SD1	0.0164	0.004	3.897	0.000	[0.008, 0.025]
AR4	−0.1293	0.069	−1.877	0.061	[−0.264, 0.006]
DR4	−0.0966	0.062	−1.568	0.117	[−0.217, 0.024]
SampEn	0.4543	0.205	2.216	0.027	[0.053, 0.856]
LF/HF	0.1009	0.008	12.789	0.000	[0.085, 0.116]

Symmetric Variables: In the N2–N3 transition analysis (see Table 6), SDNN, SD1, SampEn, and LF/HF are statistically significant predictors. SDNN has a negative coefficient (−0.0150, p=0.002), indicating that higher values reduce the probability of transitioning from N2 to N3 sleep. In contrast, SD1 (0.0164, p<0.001), SampEn (0.4543, p=0.027), and LF/HF (0.1009, p<0.001) have positive coefficients, suggesting that higher values increase the likelihood of this transition.

Asymmetric Variables: No asymmetric variables were statistically significant in this analysis. Asymmetric variables that remained in the model were AR4 and DR4.

#### 3.2.6. N3 Stage

**Table 7:** GEE regression results, N3 stage.

	Coef	Std Err	z	*p* > |z|	[0.025, 0.975]
Intercept	−1.0665	0.764	−1.396	0.163	[−2.564, 0.431]
SDNN	−0.0092	0.008	−1.182	0.237	[−0.025, 0.006]
SD1	0.0060	0.006	0.962	0.336	[−0.006, 0.018]
DR4	−0.1895	0.088	−2.146	0.032	[−0.363, −0.016]
$AR_{MAX}$	−0.1666	0.082	−2.023	0.043	[−0.328, −0.005]
$H_{AR}$	−0.4781	0.399	−1.197	0.231	[−1.261, 0.305]
SampEn	0.4975	0.185	2.692	0.007	[0.135, 0.860]

Symmetric Variables: In the N3 stage analysis (see Table 7), SampEn is a statistically significant predictor with a positive coefficient (0.4975, p=0.007), indicating that higher values significantly increase the probability of a segment being classified as the N3 stage. Other symmetric variables in the model were SDNN and SD1.

Asymmetric Variables: Among asymmetric variables, DR4 (−0.1895, p=0.032) and $AR_{MAX}$ (−0.1666, p=0.043) have statistically significant negative coefficients, indicating that higher values reduce the probability of N3 stage identification. The asymmetric variable $H_{AR}$ was also included in the model.

#### 3.2.7. N3–N2 Transition

**Table 8:** GEE regression results, N3–N2 transition.

	Coef	Std Err	z	*p* > |z|	[0.025, 0.975]
Intercept	−3.4785	0.209	−16.609	0.000	[−3.889, −3.068]
SD1	−0.0240	0.012	−2.050	0.040	[−0.047, −0.001]
$SD1_{a}$	0.0348	0.016	2.130	0.033	[0.003, 0.067]
$pnn30_{acc}$	0.0184	0.012	1.487	0.137	[−0.006, 0.043]

Symmetric Variables: In the N3–N2 transition analysis (see Table 8), SD1 is a statistically significant predictor with a negative coefficient (−0.0240, p=0.040), indicating that higher values reduce the probability of transitioning from N3 to N2 sleep.

Asymmetric Variables: Among asymmetric variables, $SD1_{a}$ is statistically significant with a positive coefficient (0.0348, p=0.033), suggesting that higher values increase the likelihood of this transition. The asymmetric variable $pnn30_{\mathrm{acc}}$ was also included in the model.

#### 3.2.8. N2–R Transition

**Table 9:** GEE regression results, N2–R transition.

	Coef	Std Err	z	*p* > |z|	[0.025, 0.975]
Intercept	−5.3598	0.846	−6.333	0.000	[−7.019, −3.701]
$AR_{MAX}$	−0.2430	0.153	−1.584	0.113	[−0.544, 0.058]
$DR_{MAX}$	0.2123	0.136	1.564	0.118	[−0.054, 0.478]
$H_{DR}$	2.4855	1.083	2.295	0.022	[0.363, 4.608]
$H_{AR}$	−1.5457	0.860	−1.798	0.072	[−3.231, 0.139]
SampEn	0.3578	0.186	1.923	0.055	[−0.007, 0.723]

Symmetric Variables: No symmetric variables were statistically significant in this analysis (see Table 9). The only symmetric variable that was included in the model was SampEn.

Asymmetric Variables: Among asymmetric variables, $H_{DR}$ is statistically significant with a positive coefficient (2.4855, p=0.022), indicating that higher values significantly increase the probability of transitioning from N2 to REM sleep. Other asymmetric variables in the model were $AR_{\mathrm{MAX}}$, $DR_{\mathrm{MAX}}$, and $H_{AR}$.

#### 3.2.9. R–N2 Transition

**Table 10:** GEE regression results, R–N2 transition.

	Coef	Std Err	z	*p* > |z|	[0.025, 0.975]
Intercept	−5.8863	0.458	−12.862	0.000	[−6.783, −4.989]
SD1	−0.0584	0.046	−1.276	0.202	[−0.148, 0.031]
$SD1_{d}$	0.0775	0.058	1.332	0.183	[−0.037, 0.191]
$AR_{MAX}$	−0.2505	0.110	−2.277	0.023	[−0.466, −0.035]
$DR_{MAX}$	0.1279	0.052	2.450	0.014	[0.026, 0.230]
$H_{DR}$	2.4818	0.595	4.169	0.000	[1.315, 3.649]

Symmetric Variables: No symmetric variables were statistically significant in this analysis (see Table 10). The symmetric variable SD1 was included in the model.

Asymmetric Variables: Among asymmetric variables, $AR_{\mathrm{MAX}}$ has a statistically significant negative coefficient (−0.2505, p=0.023), indicating that higher values reduce the probability of transitioning from REM to N2 sleep. Conversely, $DR_{\mathrm{MAX}}$ (0.1279, p=0.014) and $H_{DR}$ (2.4818, p<0.001) have statistically significant positive coefficients, suggesting that higher values increase the likelihood of this transition. The asymmetric variable $SD1_{d}$ was also included in the model.

#### 3.2.10. R Stage

**Table 11:** GEE regression results, R stage.

	Coef	Std Err	z	*p* > |z|	[0.025, 0.975]
Intercept	−2.9060	0.349	−8.337	0.000	[−3.589, −2.223]
SDNN	0.0165	0.004	4.694	0.000	[0.010, 0.023]
SD1	−0.0785	0.043	−1.827	0.068	[−0.163, 0.006]
$SD1_{d}$	0.0887	0.054	1.649	0.099	[−0.017, 0.194]
pnn30	−0.0119	0.012	−0.964	0.335	[−0.036, 0.012]
SampEn	0.1863	0.148	1.256	0.209	[−0.104, 0.477]

Symmetric Variables: In the R stage analysis (see Table 11), SDNN is a statistically significant predictor with a positive coefficient (0.0165, p<0.001), indicating that higher values significantly increase the probability of a segment being classified as the R stage. Other symmetric variables in the model were SD1, SampEn, and pnn30.

Asymmetric Variables: No asymmetric variables were statistically significant in this analysis. The asymmetric variable $SD1_{d}$ was included in the model.

#### 3.2.11. N3–R Transition

**Table 12:** GEE regression results, N3–R transition.

	Coef	Std Err	z	*p* > |z|	[0.025, 0.975]
Intercept	−6.9436	1.091	−6.364	0.000	[−9.082, −4.805]
$AR_{MAX}$	−0.4793	0.321	−1.493	0.136	[−1.109, 0.150]
$H_{DR}$	3.7051	2.091	1.772	0.076	[−0.394, 7.804]

Symmetric Variables: No symmetric variables were were left in the model (see Table 12).

Asymmetric Variables: No asymmetric variables were statistically significant in this analysis. The asymmetric variables in the model were $AR_{\mathrm{MAX}}$ and $H_{DR}$.

#### 3.2.12. W Stage (Waking)

**Table 13:** GEE regression results, W stage.

	Coef	Std Err	z	*p* > |z|	[0.025, 0.975]
Intercept	0.2604	0.823	0.316	0.752	[−1.353, 1.874]
SDNN	0.0040	0.005	0.792	0.429	[−0.006, 0.014]
SD1	−0.0352	0.020	−1.753	0.080	[−0.075, 0.004]
$SD1_{a}$	0.0506	0.028	1.804	0.071	[−0.004, 0.106]
$pnn30_{dec}$	−0.0351	0.021	−1.679	0.093	[−0.076, 0.006]
DR4	0.1820	0.054	3.373	0.001	[0.076, 0.288]
$AR_{MAX}$	0.2974	0.082	3.639	0.000	[0.137, 0.458]
$H_{DR}$	−1.9586	0.663	−2.955	0.003	[−3.258, −0.660]
$H_{AR}$	−0.4702	0.266	−1.768	0.077	[−0.991, 0.051]
SampEn	−0.2381	0.191	−1.248	0.212	[−0.612, 0.136]
LF/HF	−0.1537	0.047	−3.291	0.001	[−0.245, −0.062]

Symmetric Variables: In the W stage analysis (see Table 13), LF/HF is a statistically significant predictor with a negative coefficient (−0.1537, p=0.001), indicating that higher values significantly reduce the probability of a segment being classified as the W stage. Other symmetric variables in the model were SDNN, SD1, and SampEn.

Asymmetric Variables: Among asymmetric variables, DR4 (0.1820, p=0.001), $AR_{\mathrm{MAX}}$ (0.2974, p<0.001), and $H_{DR}$ (−1.9586, p=0.003) are statistically significant predictors. DR4 and $AR_{\mathrm{MAX}}$ have positive coefficients, indicating that higher values increase the probability of the W stage, while $H_{DR}$ has a negative coefficient, suggesting that higher values reduce this probability. Other asymmetric variables in the model were $SD1_{a}$, $pnn30_{\mathrm{dec}}$, and $H_{AR}$.

### 3.3. Predictive Modelling Results

This subsection evaluates the performance of predictive models for various sleep stages and transitions. We present confusion matrices and performance metrics (AUC, accuracy, precision, recall, and F1 score) for each stage and transition, highlighting the effectiveness and areas for improvement of the models. The models, as indicated in Section 2, were the same models as described in the previous section, but the predictions are calculated for the test set.

#### 3.3.1. N1 Stage

**Table 14:** Results of predictive modeling: confusion matrix and performance metrics for N1 stage detection.

Confusion Matrix							Performance Metrics	
		**Predicted**					AUC:	0.74
		Negative	Positive				Accuracy:	0.68
**Actual**	Negative	407	218				Precision:	0.55
	Positive	105	270				Recall:	0.72
							F1 Score:	0.63

The model demonstrated in Table 14 achieves an AUC of 0.74, indicating a fair ability to discriminate between sleep stage N1 and other stages. It shows a recall rate of 72%, indicating effectiveness in identifying positive cases of sleep stage N1. A precision of 55% suggests a notable number of false positives, where other sleep stages are misclassified as N1. The overall accuracy is 68%, indicating reasonable performance with room for improvement. An F1 score of 0.63 shows balanced precision and recall.

#### 3.3.2. N2 Stage

**Table 15:** Results of predictive modeling: confusion matrix and performance metrics for N2 stage detection.

Confusion Matrix							Performance Metrics	
		**Predicted**					AUC:	0.85
		Negative	Positive				Accuracy:	0.77
**Actual**	Negative	365	113				Precision:	0.78
	Positive	113	409				Recall:	0.78
							F1 Score:	0.78

The model demonstrated in Table 15 attains an AUC of 0.85, signifying a strong discriminative ability in differentiating sleep stage N2 from other stages. A recall rate of 78% demonstrates effective identification of true positive N2 cases. The precision of 78% indicates a balanced proportion of correct positive predictions, suggesting fewer false positives. An overall accuracy of 77% reflects solid model performance. An F1 score of 78% shows balanced precision and recall.

#### 3.3.3. N3 Stage

**Table 16:** Results of predictive modeling: confusion matrix and performance metrics for N3 stage detection.

Confusion Matrix							Performance Metrics	
		**Predicted**					AUC:	0.89
		Negative	Positive				Accuracy:	0.81
**Actual**	Negative	433	107				Precision:	0.78
	Positive	86	374				Recall:	0.81
							F1 Score:	0.79

The model demonstrated in Table 16 achieves an AUC of 0.89, demonstrating excellent discriminative power in distinguishing the N3 sleep stage from other stages. A recall rate of 81% indicates effective identification of true positive N3 cases. The precision of 78% suggests a good balance between correctly predicted positive instances and false positives. An overall accuracy of 81% reflects robust performance across the dataset. An F1 score of 79% shows balanced precision and recall.

#### 3.3.4. R Stage

**Table 17:** Results of predictive modeling: confusion matrix and performance metrics for R stage detection.

Confusion Matrix							Performance Metrics	
		**Predicted**					AUC:	0.61
		Negative	Positive				Accuracy:	0.59
**Actual**	Negative	277	233				Precision:	0.57
	Positive	178	312				Recall:	0.64
							F1 Score:	0.60

The model demonstrated in Table 17 achieves an AUC of 0.61, indicating a modest capacity to distinguish the R sleep stage from other stages. A recall rate of 64% shows that the model moderately captures true positive instances of the R stage. The precision of 57% reveals a considerable number of false positives, meaning other sleep stages are frequently misclassified as the R stage. An overall accuracy of 59% reflects average performance, suggesting significant room for improvement. The F1 score is 60%.

#### 3.3.5. W Stage

**Table 18:** Results of predictive modeling: confusion matrix and performance metrics for W stage detection.

Confusion Matrix							Performance Metrics	
		**Predicted**					AUC:	0.87
		Negative	Positive				Accuracy:	0.80
**Actual**	Negative	453	107				Precision:	0.76
	Positive	95	345				Recall:	0.78
							F1 Score:	0.77

With an AUC of 0.87, the model in Table 18 demonstrates a strong ability to distinguish the W stage from other sleep stages. A recall rate of 78% indicates effective identification of true positive cases for the W stage. The precision of 76% suggests a balanced classification with relatively few false positives. An overall accuracy of 80% reflects solid performance across all classes. The F1 score is 77%.

#### 3.3.6. N2–N3 Transition

**Table 19:** Results of predictive modeling: confusion matrix and performance metrics for N2–N3 transition detection.

Confusion Matrix							Performance Metrics	
		**Predicted**					AUC:	0.80
		Negative	Positive				Accuracy:	0.71
**Actual**	Negative	498	222				Precision:	0.49
	Positive	66	214				Recall:	0.76
							F1 Score:	0.60

The model in Table 19 achieves an AUC of 0.80, indicating a strong ability to distinguish the N2–N3 transition from other sleep stages. A recall rate of 76% demonstrates effective identification of true positive cases for the N2–N3 transition. However, a precision of 49% suggests a substantial number of false positives, meaning other stages are frequently misclassified as the N2–N3 transition. An overall accuracy of 71% reflects reasonable performance but indicates room for improvement. The F1 score is 60%.

#### 3.3.7. N2–R Transition

**Table 20:** Results of predictive modeling: confusion matrix and performance metrics for N2–R transition detection.

Confusion Matrix							Performance Metrics	
		**Predicted**					AUC:	0.85
		Negative	Positive				Accuracy:	0.76
**Actual**	Negative	297	120				Precision:	0.80
	Positive	116	467				Recall:	0.80
							F1 Score:	0.80

The model shown in Table 20 attains an AUC of 0.85, indicating a strong capacity to differentiate the N2–R transition from other sleep stages. A recall rate of 80% signifies effective detection of true positive instances for this transition. The precision of 80% suggests a balanced performance with a low rate of false positives. An overall accuracy of 76% reflects solid predictive capabilities. The F1 score is 80%.

#### 3.3.8. N3–N2 Transition

In this case, there were not enough data points in the test group, so the results below are for the training group.

**Table 21:** Results of predictive modeling: confusion matrix and performance metrics for N3–N2 transition detection.

Confusion Matrix							Performance Metrics	
		**Predicted**					AUC:	0.63
		Negative	Positive				Accuracy:	0.60
**Actual**	Negative	375	254				Precision:	0.47
	Positive	145	226				Recall:	0.61
							F1 Score:	0.53

The model in Table 21 shows a recall rate of 61%, indicating moderate effectiveness in identifying positive cases of the N3–N2 transition. A precision of 47% suggests a significant number of false positives, where other sleep stages are misclassified as the N3–N2 transition. The overall accuracy is 60%, indicating reasonable performance with room for improvement. An F1 score of 53% shows balanced precision and recall.

#### 3.3.9. N3–R Transition

**Table 22:** Results of predictive modeling: confusion matrix and performance metrics for N3–R transition detection.

Confusion Matrix							Performance Metrics	
		**Predicted**					AUC:	0.72
		Negative	Positive				Accuracy:	0.67
**Actual**	Negative	272	163				Precision:	0.71
	Positive	167	398				Recall:	0.70
							F1 Score:	0.71

With an AUC of 0.72, the model from Table 22 exhibits a moderate capability to differentiate the N3–R transition from other sleep stages. A recall rate of 70% signifies effective detection of true positive cases for this transition. The precision of 71% indicates a well-balanced classification, resulting in relatively few false positives. An overall accuracy of 67% reflects acceptable performance, though there is room for improvement. The F1 score is 71%.

#### 3.3.10. R–N2 Transition

**Table 23:** Results of predictive modeling: confusion matrix and performance metrics for R–N2 transition detection.

Confusion Matrix							Performance Metrics	
		**Predicted**					AUC:	0.80
		Negative	Positive				Accuracy:	0.72
**Actual**	Negative	515	229				Precision:	0.47
	Positive	55	201				Recall:	0.79
							F1 Score:	0.59

The model demonstrated in Table 23 achieves an AUC of 0.80, demonstrating a solid capacity to differentiate the R–N2 transition from other sleep stages. The model shows a recall rate of 79%, indicating effectiveness in identifying positive cases of the R–N2 transition. A precision of 47% suggests a significant number of false positives, where other sleep stages are misclassified as the R–N2 transition. The overall accuracy is 72%, indicating reasonable performance with room for improvement. The F1 score is 59%.

#### 3.3.11. W–N1 Transition

**Table 24:** Results of predictive modeling: confusion matrix and performance metrics for W–N1 transition detection.

Confusion Matrix							Performance Metrics	
		**Predicted**					AUC:	0.83
		Negative	Positive				Accuracy:	0.74
**Actual**	Negative	535	210				Precision:	0.49
	Positive	52	203				Recall:	0.80
							F1 Score:	0.61

The model in Table 24 achieves an AUC of 0.83, demonstrating a robust ability to distinguish the W–N1 transition from other sleep stages. The model shows a recall rate of 80%, indicating effectiveness in identifying positive cases of the W–N1 transition. A precision of 49% suggests a significant number of false positives, where other sleep stages are misclassified as the W–N1 transition. The overall accuracy is 74%, indicating reasonable performance with room for improvement. The F1 score is 61%.

## 4. Discussion

This study investigated the potential of enhancing the traditional symmetric variability and complexity parameters derived from heart rate variability and complexity with asymmetric variability and complexity measures to detect and differentiate sleep stages. It should be stressed that this paper does not consider a single model, but a separate model for each stage and transition, so the statistical part of the analysis can only be treated as exploratory. The results demonstrate the efficacy of using HRA metrics combined with traditional HRV and entropy measures, to accurately identify sleep stages and transitions between them. In all cases, asymmetric variables had an independent predictive value in detecting the sleep stages and transitions between them. The variables that most often survived in the backward selection process were asymmetric entropies, $H_{DR}$ and $H_{AR}$. The performance of the models as predictive tools was also good, considering the very diverse nature of the study group, which included subjects of varying ages and health conditions.

### 4.1. Sleep Stage Classification and HRV

Three classical HRV variables were used in the present study: SDNN, SD1, and LF/HF. Another symmetric variable, pnn30, that was used in this paper, is closely related to a popular HRV descriptor, namely pnn50 [17].

The variables SDNN and LF/HF lead to the best results in several models. For SDNN, it shows a significant positive effect in the transitions N1–N2 and W–N1 and the R stage. In the N1–N2 transition, an increase in SDNN increases the likelihood of transitioning from N1 to N2. Similarly, in the W–N1 transition, higher SDNN values increase the probability of transitioning from wakefulness to N1. In the R stage, higher SDNN increases the likelihood of detecting REM sleep.

For LF/HF, the best result is observed in the N2–N3 transition, where an increase in LF/HF increases the likelihood of transitioning from N2 to N3. Additionally, in the N2 stage, higher LF/HF increases the probability of detecting the N2 stage.

Our findings reinforce the significance of HRV in sleep stage detection, aligning with previous research. For instance, the higher high-frequency components and lower low-frequency components during non-REM sleep stages reflect heightened parasympathetic activity, as noted in [19,20]. Conversely, the increased LF/HF ratio during REM sleep indicates elevated sympathetic activity, supporting the autonomic shifts reported in [21].

### 4.2. Sleep Stage Classification Entropy Measures

SampEn leads to very good results in detecting several stages and transitions. Notably, it has significant negative and positive effects depending on the model.

For the N1 stage, a decrease in SampEn increases the likelihood of detecting the N1 stage. This suggests that lower SampEn values are associated with a higher probability of detecting N1 sleep.

In the N2–N3 transition, an increase in SampEn significantly increases the probability of transitioning from N2 to N3.

Similarly, in the N3 stage, higher SampEn values increase the likelihood of detecting the N3 stage.

While SampEn also appears in other models, such as the N2–R transition and the R stage, these results are not statistically significant and do not strongly contribute to detecting those stages or transitions.

The significant association of SampEn with deeper sleep stages, as seen in our results, is consistent with previous research by [25,26]. The decrease in SampEn during transitions to deeper sleep stages reflects the reduced complexity and increased regularity of physiological signals, reinforcing the notion of a stable sleep state—this also further supports the results of [25,26].

### 4.3. Sleep Stage Classification with HRA and Symmetric Parameters

Our results indicate that HRA, especially its complexity and runs-based aspects, significantly improves the statistical properties of inferential models for sleep analysis as well as improving the predictive properties of these models. Notably, HRA descriptors like $H_{DR}$ and $H_{AR}$ played a pivotal role in improving model performance, particularly in detecting specific sleep stages and transitions.

Among the asymmetric variables analyzed, $H_{DR}$ significantly increased the probability of identifying the N2 stage and played a crucial role in both the N2–R and R–N2 transitions. Similarly, $H_{AR}$ was highly effective in increasing the probability of the N2 stage and in facilitating the transition from N1 to N2 sleep. In addition to $H_{DR}$ and $H_{AR}$, $AR_{MAX}$ demonstrated a strong influence on the identification of the N2 and W stages, significantly reducing the likelihood of the N2 stage while increasing the probability of the W stage. Moreover, $AR_{MAX}$ played a significant role in the R–N2 transition, where its reduced value lowered the probability of transitioning from REM to N2 sleep. Lastly, the drop in DR4 was most effective in reducing the probability of both the N2 and N3 sleep stages.

Another noteworthy observation is that even when asymmetric variables did not directly lead to better predictions (as in cases where their presence was not statistically significant), they stabilized the models and reduced the Akaike Information Criterion (AIC), highlighting their value in predictive modeling. Runs-based parameters, in particular, were the most effective in predicting and differentiating between sleep stages and transitions, offering a granularity not provided by symmetric measures.

The differentiation of sleep stages based on the asymmetrical contributions of heart rate decelerations and accelerations aligns with previous studies by Guzik et al. [33] and Apnea et al. [34]. The reduced number of short deceleration and acceleration runs in patients with severe OSA, coupled with the increased occurrence of longer runs, highlights the impact of sleep disorders on heart rate dynamics. These findings are consistent with research by Herzig et al. [20] and Tsunoda et al. [21], who reported altered HRV patterns in individuals with sleep disorders.

### 4.4. The Best Models

Six models stand out as particularly effective and potentially useful. These models include three stages and three transitions. The symmetric and asymmetric parameters used in constructing these models exhibit an interesting pattern.

The models with the best performance for the sleep stages are the models for the deeper sleep stages, namely N2 and N3, and the model for the wafeful stage, W. The models for these stages have AUCs of 0.85, 0.89, and 0.87, respectively. The other performance metrics for these stages are also good, for example accuracy (77%, 81%, and 80%, respectively) and recall (78%, 81%, and 78% respectively). The analysis of the structure of the statistical models leads to the conclusion that in all these models, both symmetric and asymmetric variables are important. In all of them, the complexity parameters, both symmetric (SampEn) and asymmetric ($H_{DR},H_{AR})$, are essential.

The models with the best performance for transitions are the transitions from N2 to REM and back (N2–R, R–N2) and falling asleep (W–N1). The AUC for these transitions are 0.85, 0.80, and 0.83, respectively, and the other performance metrics, like accuracy (76%, 72%, and 74%, respectively) and recall (80%, 79%, and 80%, respectively), are also good. However, the structure of the statistical models is different. All three are dominated by asymmetric parameters, with SampEn in the N2–R model, SD1 in the R–N2 model, and SDNN in W–N1 as the sole representatives of symmetric parameters. All of these models contained $AR_{MAX}$ and either $H_{AR}$ or $H_{DR}$ or both.

### 4.5. GEE in Sleep Stages Studies

In this paper, we proposed the use of the Generalized Estimating Equation (GEE) in sleep studies, as it is particularly well-suited for analyzing clustered data, which naturally occurs in sleep research, where each patient represents a cluster. We developed the best statistical models using GEE and applied them to machine learning analyses. By accounting for the internal correlations within each cluster (patient), GEE makes both statistical inference and machine learning methods explainable and more manageable. The results obtained through GEE in our study align with those found by other authors using different statistical or machine learning methods, reinforcing the validity of our approach. We believe that GEE represents the best of both worlds—statistical inference and machine learning—when applied to the study of sleep stages and the transitions between them.

### 4.6. Clinical, Research, and Engineering Implications

The integration of Heart Rate Asymmetry (HRA) with traditional measures such as HRV and entropy in sleep stage classification presents significant clinical and research implications. Based on the results and discussion presented, we conclude that HRA provides information about sleep stages that is both independent of and complementary to established metrics such as SDNN, the LF/HF ratio, and SampEn. This is evident from the multivariate statistical models presented in this paper.

The practicality of using HRA in portable and mobile devices for sleep analysis cannot be overstated. Since HRA can be derived from the most accessible and cost-effective signals, such as the ECG or RR interval time series, it allows for real-time data collection without the need to store complete ECG recordings.

Sleep stages and transitions such as deep sleep stages (N2 and N3), falling asleep (W–N1 transition), and the beginning and end of REM sleep (N2–R and R–N2 transitions) are crucial for applications in clinical diagnostics, research, engineering, and mobile health monitoring. The models developed for these stages and transitions were actually the best among all models discussed in the present paper. These results could be applied in clinical practice to improve sleep disorder diagnostics, support research by providing more accurate sleep stage data, and aid engineering efforts in developing affordable mobile and portable sleep monitoring devices.

## 5. Limitations of the Study

This study, while providing insights into sleep stages and transitions, is subject to several limitations that warrant consideration.

The most important limitation of this study is the heterogeneity of the study population. The participants included individuals with a wide range of ages and various medical conditions. The inclusion of subjects with different comorbidities may introduce variability that could influence heart rate dynamics and potentially confound the interpretations of the results.

Specifically, conditions such as hypertension and OSA are known to affect autonomic nervous system function and heart rate variability [10,33,56]. Hypertension is associated with altered autonomic balance and reduced heart rate variability, while OSA leads to intermittent hypoxia and sympathetic overactivation, impacting heart rate dynamics and heart rate asymmetry [9,33]. Including hypertensive patients and those with sleep-disordered breathing patterns in one group may, therefore, mask or amplify certain features related to heart rate variability and asymmetry associated with specific sleep stages and transitions. Moreover, other conditions present in the study group, such as diabetes, stroke, and obesity, can further affect cardiovascular autonomic regulation.

Future studies should consider focusing on healthy subjects or more homogeneous groups to isolate the effects of heart rate asymmetry on sleep stages without the confounding influence of various diseases. Such an approach would help in determining whether the observed associations are inherent to the sleep stages themselves or are influenced by underlying health conditions.

Also, the sample size was relatively small, comprising only 31 polysomnographic recordings (clusters), even though each of them supplied a large number of sleep stages. This limited sample size may affect the generalizability of the findings, as it may not capture the full spectrum of variability seen in larger populations.

Additionally, while the Generalized Estimating Equation (GEE) models provided robust estimates, the exact nature of the within-recording correlations remains complex and not fully understood. Further research is needed to explore different correlation structures and their impact on model performance. Understanding these internal correlations better could improve the accuracy and applicability of the GEE models in future studies.

Acknowledging these limitations, we believe our findings contribute to sleep research. They should, however, be viewed with caution, and additional research with larger and more uniform samples is advised.

## 6. Conclusions

This study demonstrates the utility of Heart Rate Asymmetry (HRA) in sleep stage classification and transition detection, providing strong performance metrics across key stages and transitions. The incorporation of HRA parameters yielded high levels of accuracy, recall, and area under the curve (AUC) in statistical and predictive models.

For stage detection, models utilizing $H_{DR}$ and $H_{AR}$ achieved an AUC of 0.85, a recall of 78%, and an accuracy of 77% for stage N2, as well as an AUC of 0.89, an accuracy of 81%, and a recall of 81% for stage N3. The model for detecting the waking state (W) had an AUC of 0.87, accuracy of 80%, and a recall of 78%. In the structure of the statistical models for these stages, symmetric and asymmetric parameters are equally important.

For transitions, models with $H_{DR}$ demonstrated strong predictive performance in N2–R and R–N2 transitions, achieving an AUC of 0.85, an accuracy of 76%, and a recall of 80% and an AUC of 0.80, an accuracy of 72%, and a recall 79%, respectively. Additionally, a model for detecting the W–N1 transition, i.e., falling asleep, had an AUC of 0.83, an accuracy of 77%, and a recall of 80%. The structure of the statistical models tor transitions is clearly dominated by asymmetric variables.

These findings suggest that HRA measures can offer valuable metrics for sleep stage classification and transition detection, complementing traditional heart rate variability methods. This approach could enhance sleep disorder diagnostics, support research with more accurate sleep stage data, and aid in developing portable, affordable sleep monitors.

## Figures and Tables

**Table 1:** The overall distribution of sleep stages and transitions in the analyzed recordings.

	Number of Stages Across All Recordings	Percentage of All Recordings
N1 stage	138	4.0%
N2 stage	868	25.17%
N3 stage	328	9.51%
R stage	270	7.83%
W stage	680	19.72%
transition N1–N2	264	7.65%
transition N2–N3	127	3.68%
transition N2–R	55	1.59%
transition N3–N2	112	3.25%
transition N3–R	10	0.29%
transition R–N2	53	1.54%
transition W–N1	544	15.77%

**Table 2:** GEE regression results, N1 stage.

	Coef	Std Err	z	*p* > |z|	[0.025, 0.975]
Intercept	−3.5554	0.709	−5.015	0.000	[−4.945, −2.166]
SDNN	−0.0236	0.009	−2.693	0.007	[−0.041, −0.006]
$SD1_{a}$	0.0322	0.012	2.682	0.007	[0.009, 0.056]
$pnn30_{acc}$	0.0246	0.019	1.271	0.204	[−0.013, 0.063]
$DR_{MAX}$	0.0863	0.053	1.632	0.103	[−0.017, 0.190]
$H_{DR}$	0.9044	0.730	1.239	0.215	[−0.527, 2.335]
SampEn	−0.4135	0.160	−2.581	0.010	[−0.728, −0.099]

**Table 3:** GEE regression results, N2 stage.

	Coef	Std Err	z	*p* > |z|	[0.025, 0.975]
Intercept	−1.8036	0.579	−3.116	0.002	[−2.938, −0.669]
SDNN	−0.0054	0.003	−1.581	0.114	[−0.012, 0.001]
SD1	−0.0317	0.021	−1.511	0.131	[−0.073, 0.009]
$SD1_{d}$	0.0436	0.025	1.719	0.086	[−0.006, 0.093]
pnn30	0.0102	0.008	1.317	0.188	[−0.005, 0.025]
DR4	−0.1330	0.045	−2.985	0.003	[−0.220, −0.046]
$AR_{MAX}$	−0.2090	0.061	−3.419	0.001	[−0.329, −0.089]
$H_{DR}$	1.2553	0.607	2.069	0.039	[0.066, 2.445]
$H_{AR}$	0.4440	0.161	2.754	0.006	[0.128, 0.760]
SampEn	0.2418	0.137	1.770	0.077	[−0.026, 0.509]
LF/HF	0.0835	0.015	5.464	0.000	[0.054, 0.113]

**Table 4:** GEE regression results, N1–N2 transition.

	Coef	Std Err	z	*p* > |z|	[0.025, 0.975]
Intercept	−3.1716	0.218	−14.542	0.000	[−3.599, −2.744]
SDNN	0.0088	0.004	2.429	0.015	[0.002, 0.016]
$SD1_{d}$	−0.0067	0.004	−1.651	0.099	[−0.015, 0.001]
$H_{AR}$	0.4436	0.197	2.257	0.024	[0.058, 0.829]

**Table 5:** GEE regression results, W–N1 transition.

	Coef	Std Err	z	*p* > |z|	[0.025, 0.975]
Intercept	−2.7905	0.331	−8.433	0.000	[−3.439, −2.142]
SDNN	0.0135	0.003	4.097	0.000	[0.007, 0.020]
SD1	−0.4619	0.270	−1.710	0.087	[−0.991, 0.067]
$SD1_{a}$	0.3156	0.182	1.733	0.083	[−0.041, 0.672]
$SD1_{d}$	0.3268	0.200	1.637	0.102	[−0.065, 0.718]
$AR_{MAX}$	0.0632	0.032	1.951	0.051	[−0.000, 0.127]
$H_{AR}$	0.3915	0.246	1.592	0.111	[−0.090, 0.873]

## Data Availability

The data presented in this study are available on request from the corresponding author.
